# The Incidence and Outcomes of Late-Term Pregnancy

**DOI:** 10.7759/cureus.33550

**Published:** 2023-01-09

**Authors:** Amene Ranjbar, Vahid Mehrnoush, Fatemeh Darsareh, Faranak Pariafsay, Malihe Shirzadfardjahromi, Mitra Shekari

**Affiliations:** 1 Fertility and Infertility Research Center, Hormozgan University of Medical Sciences, Bandar Abbas, IRN; 2 Urology Department, Northern Ontario School of Medicine, Thunder Bay, CAN; 3 Mother and Child Welfare Research Center, Hormozgan University of Medical Sciences, Bandar Abbas, IRN

**Keywords:** childbirth, pregnancy, neonatal outcome, maternal outcome, late-term pregnancy

## Abstract

Background: Little is known about the outcomes of late-term pregnancy. In this study, we aim to assess the incidence and adverse prenatal outcomes associated with late-term pregnancy.

Methods: We retrospectively assessed all singleton pregnant mothers who gave birth at Khalij-e-Fars Hospital in Bandar Abbas, Iran, between January 2020 and 2022. All preterm and post-term deliveries were excluded. Mothers were divided into two groups: late-term mothers (41 0/7-41 6/7 weeks of gestation) and term mothers (37 0/7-40 6/7 weeks of gestation). Demographic factors, obstetric factors, maternal comorbidities, and prenatal outcomes were extracted from the electronic data of each mother. The incidence of late-term births was calculated. The chi-squared test was used to compare differences between the groups. Logistic regression models were used to assess the association of prenatal outcome with late-term pregnancy.

Results: There were 8,888 singleton deliveries during the study period, and 1,269 preterm and post-term pregnancies were ruled out. Of the 7,619 deliveries, 309 (4.1%) were late-term, while 7,310 (95.9%) were term. There were no sociodemographic differences between term and late-term mothers. The late-term group had a higher prevalence of primiparous mothers, and the term group had a higher prevalence of diabetes. Late-term mothers had a higher risk of macrosomia (adjusted odds ratio (aOR): 2.24 (95% confidence interval (CI): 1.34-3.01)), meconium amniotic fluid (aOR: 2.32 (95% CI: 1.59-3.14)), and fetal distress (aOR: 2.38 (95% CI: 1.54-2.79)). When compared to term pregnancy, the risk of low birth weight (LBW) was lower in late-term pregnancy (aOR: 0.69 (95% CI: 0.36-0.81)).

Conclusions: Late-term pregnancy was found to be more likely to be associated with macrosomia, meconium amniotic fluid, and fetal distress, but serious maternal and neonatal adverse events were comparable to term pregnancy.

## Introduction

Gestation lasts an average of 40 weeks (280 days) from the first day of the last menstrual period to the estimated date of delivery in singleton pregnancies [1]. Previously, the period from three weeks before the estimated date of delivery to two weeks after the estimated date of delivery was considered “term,” with the expectation that neonatal outcomes from deliveries during this interval would be uniform and good. However, research has increasingly revealed that neonatal outcomes, particularly respiratory morbidity, vary depending on the timing of delivery, even within this five-week gestational age range [2]. The risk of adverse neonatal outcomes is lowest in uncomplicated pregnancies delivered between 39 0/7 and 40 6/7 weeks of gestation [3]. In low-risk females, it is being debated whether to induce labor at 41 weeks + 0 days or to allow the pregnancy to continue until 42 weeks + 0 days. Post-term pregnancy is linked to poor perinatal and maternal outcomes [4]. However, little is known about the link between late-term pregnancy and poor perinatal and maternal outcomes. In this study, we aim to assess the incidence and adverse prenatal outcomes associated with late-term pregnancy.

This article was previously posted to the Research Square preprint server on July 22, 2022.

## Materials and methods

This study complies with the Declaration of Helsinki and was performed according to ethics committee approval. The Ethics and Research Committee of Hormozgan University of Medical Sciences approved the study. The records of all patients who provided informed consent for using their data for research purposes were analyzed. In cases of illiteracy, their legal guardians provided informed consent. Statistical analysis was performed with patient anonymity following ethics committee regulations. We retrospectively assessed all singleton pregnant mothers who gave birth at Khalij-e-Fars Hospital (a tertiary hospital) in Bandar Abbas, Iran, between January 1st, 2020, and January 1st, 2022. All preterm and post-term deliveries were excluded from the study. Using electronic patient records, data were extracted by trained collectors from the “Iranian Maternal and Neonatal Network (IMaN Net),” a valid national system. Mothers were divided into two groups based on gestational age at birth: late-term mothers and term mothers. Term pregnancy was defined as 37 0/7 weeks to 40 6/7 weeks of gestation, and late-term pregnancy was defined as 41 0/7 weeks to 41 6/7 weeks of gestation [5].

Demographic factors (age, educational level, living residency, medical insurance, access to prenatal care facilities, and smoking status), obstetrical factors (parity, infertility, use of assisted reproductive technology (ART), newborn sex, abnormal placentation (previa/acreta), oligohydramnios, and polyhydramnios), maternal comorbidities (diabetes mellitus, chronic hypertension, preeclampsia, cardiovascular disease, thyroid dysfunction, drug addiction, hepatitis, anemia, and COVID-19 at the time of admission), and prenatal outcomes (onset of labor, mode of delivery, placenta abruption, intrauterine growth restriction (IUGR), intrauterine fetal death (IUFD), shoulder dystocia, perineal laceration, post-partum hemorrhage, intensive care unit (ICU) admission, and maternal death, low birth weight (LBW), macrosomia (newborn weight of more than 4,000 grams), congenital malformation, meconium amniotic fluid, fetal distress (abnormal fetal heart pattern or rate), asphyxia, childbirth trauma (clavicle fracture, Erb’s palsy, and Klumpke’s palsy), need for resuscitation, neonatal intensive care unit (NICU) admission, and newborn death) were extracted from electronic data of each mother.

The Statistical Package for the Social Sciences (SPSS) version 25 (IBM SPSS Statistics, Armonk, NY, USA) was used to examine the data. Categorical variables are presented as numbers and frequencies (%). The chi-squared test was used to compare differences between the groups for categorical variables. Logistic regression models were used to assess the association of prenatal outcome with late-term pregnancy. The result was presented as odds ratio (OR) or adjusted odds ratio (aOR) after adjusting for confounders and 95% confidence interval (CI). P < 0.05 was considered statistically significant, and all statistical tests were two-tailed.

## Results

There were 8,888 singleton deliveries during the study period, and 1,269 preterm and post-term pregnancies were ruled out. Of the 7,619 pregnancies, 309 (4.1%) were late-term, while 7,310 (95.9%) were term. Table 1 shows the sociodemographic differences between term and late-term mothers, with no statistically significant differences between the two groups.

**Table 1:** Demographic differences of the study population based on gestational age Data are presented as numbers (%).

Demographic characteristics	Term (n = 7,310)	Late term (n = 309)	P value
Age (years)			0.121
13-19	147 (2)	5 (1.6)	
20-34	6,021 (82.4)	260 (84.1)	
35 and above	1,142 (15.6)	38 (12.3)	
Educational level			0.093
Illiterate	446 (6.1)	30 (9.7)	
Elementary	2,234 (30.6)	101 (32.7)	
High school/diploma	3,408 (46.6)	128 (41.4)	
Advanced	1,220 (16.7)	50 (16.2)	
Residency place			0.130
Urban	4,893 (66.9)	195 (63.1)	
Rural	2,417 (33.1)	114 (36.9)	
Access to prenatal care			0.457
Yes	7,233 (98.9)	308 (99.7)	
No	77 (1.1)	1 (0.3)	
Medical insurance			0.369
Yes	7,304 (99.9)	309 (100)	
No	6 (0.1)	0	
Smoking			0.315
Yes	18 (0.2)	0	
No	7,292 (99.8)	309 (100)	

Table 2 shows that the groups differed statistically in terms of parity and diabetes incidence. The late-term group was mostly first-time pregnant mothers. The term group had a higher prevalence of diabetes.

**Table 2:** Obstetrical and medical differences of the study population based on gestational age Data are presented as numbers (%). ART: assisted reproductive technology, COVID-19: coronavirus disease 2019

Variables	Term (n = 7,310)	Late term (n = 309)	P value
Parity			<0.001
Primiparous	2,021 (27.6)	127 (41.1)	
Multiparous (2-5)	5,107 (69.9)	170 (55)	
Grand multiparous (6 parity or more)	181 (2.5)	12 (3.9)	
Infertility			0.356
No	7,200 (98.5)	308 (99.7)	
Yes	110 (1.5)	1 (0.3)	
ART			0.669
No	7,291 (99.8)	309 (100)	
Yes	19 (0.2)	0	
Newborn sex			0.250
Male	3,724 (50.9)	164 (53.1)	
Female	3,586 (49.1)	145 (46.9)	
Abnormal placentation			0.385
No	7,287 (99.7)	309 (100)	
Yes	23 (0.3)	0	
Oligohydramnios			
No	7,253 (99.2)	309 (100)	0.249
Yes	57 (0.8)	0	
Polyhydramnios			0.883
No	7,301 (99.9)	309 (100)	
Yes	9 (0.1)	0	
Anemia			0.133
No	7,103 (97.2)	307 (99.4)	
Yes	207 (2.8)	2 (0.6)	
Cardiovascular disease			0.408
No	7,237 (99)	307 (99.4)	
Yes	73 (1)	2 (0.6)	
Drug addiction			0.104
No	7,286 (99.6)	306 (99)	
Yes	24 (0.4)	3 (1)	
Chronic hypertension			0.455
No	7,242 (99.1)	307 (99.4)	
Yes	68 (0.9)	2 (0.6)	
Preeclampsia			0.292
No	6,962 (95.2)	297 (96.1)	
Yes	348 (4.8)	12 (3.9)	
COVID-19			0.094
No	7,208 (98.6)	308 (99.7)	
Yes	102 (1.4)	1 (0.3)	
Hypothyroidism			0.324
No	6,545 (89.5)	282 (91.3)	
Yes	765 (10.5)	27 (8.7)	
Hepatitis			0.300
No	7,281 (99.6)	309 (100)	
Yes	29 (0.4)	0	
Diabetes			<0.001
No	6,221 (85.1)	306 (99.1)	
Overt	11 (0.2)	1 (0.3)	
Gestational	1,078 (14.7)	2 (0.6)	

The chi-squared test was used to compare the adverse maternal and neonatal outcomes of term and late-term mothers. As shown in Table 3, the incidence of LBW, macrosomia, meconium amniotic fluid, and fetal distress differed between groups, with late-term mothers having a higher rate of macrosomia, meconium amniotic fluid, and fetal distress and a lower rate of LBW. However, there was no statistically significant difference between groups in terms of resuscitation rate, asphyxia, or NICU admission.

**Table 3:** Maternal and neonatal outcomes of the study population based on gestational age Data are presented as numbers (%). LBW: low birth weight; IUFD: intrauterine fetal death; IUGR: intrauterine growth retardation; ICU: intensive care unit; NICU: neonatal intensive care unit

Variables	Term (n = 7,310)	Late term (n = 309)	P value
Onset of labor			<0.001
Spontaneous	4,069 (55.7)	139 (45)	
Induction	1,724 (23.6)	157 (49.2)	
Elective cesarean section	1,516 (20.7)	13 (4.2)	
Placenta abruption			0.290
No	7,142 (97.7)	304 (98.4)	
Yes	168 (2.3)	5 (1.6)	
Meconium fluid			<0.001
No	6,357 (87)	231 (74.8)	
Yes	950 (13)	78 (25.2)	
Fetal distress			<0.001
No	6,743 (92.2)	256 (82.8)	
Yes	567 (7.8)	53 (17.2)	
Method of delivery			0.421
Normal vaginal delivery	4,997 (68.4)	210 (68)	
Instrumental delivery	74 (1)	4 (1.3)	
Cesarean section	2,239 (30.6)	95 (30.7)	
Grade 3 or 4 perineal lacerations			0.813
No	7,305 (99.9)	309 (100)	
Yes	5 (0.1)	0	
Post-partum hemorrhage			0.437
No	7,185 (99.3)	304 (98.4)	
Yes	125 (1.7)	5 (1.6)	
LBW			<0.001
No	6,899 (94.3)	306 (99)	
Yes	411 (5.7)	3 (1)	
Macrosomia (more than 4,000 grams)			<0.001
No	7,158 (97.9)	280 (90.6)	
Yes	152 (2.1)	29 (9.4)	
IUGR			0.304
No	7,117 (97.4)	305 (98.7)	
Yes	191 (2.6)	4 (1.3)	
IUFD			0.678
No	7,299 (99.8)	308 (99.7)	
Yes	11 (0.2)	1 (0.3)	
Childbirth injury			0.884
No	7,293 (99.9)	309 (100)	
Yes	8 (0.1)	0	
Shoulder dystocia			0.452
No	7,252 (99.2)	306 (99)	
Yes	58 (0.8)	3 (1)	
Neonatal congenital malformation			0.976
No	7,259 (99.3)	307 (99.4)	
Yes	51 (0.7)	2 (0.6)	
Need for neonatal resuscitation			0.402
No	6,867 (93.9)	287 (92.9)	
Yes	443 (6.1)	22 (7.1)	
Newborn asphyxia			0.911
No	7,288 (99.7)	308 (99.7)	
Yes	22 (0.3)	1 (0.3)	
NICU admission			0.417
No	7,024 (96.1)	298 (96.4)	
Yes	286 (3.9)	11 (3.6)	
Newborn death			0.982
No	7,308 (100)	309 (100)	
Yes	0	0	

Table 4 shows the risk of adverse prenatal outcomes in late-term pregnancy. Late-term pregnancy was associated with LBW, macrosomia, meconium amniotic fluid, and fetal distress, according to bivariate regression analysis. After adjusting for confounders (sociodemographic factors, obstetrical factors, and maternal comorbidities), late-term mothers had a higher risk of macrosomia (aOR: 2.24 (95% CI: 1.34-3.01)), meconium amniotic fluid (aOR: 2.32 (95% CI: 1.59-3.14)), and fetal distress (aOR: 2.38 (95% CI: 1.54-2.79)). When compared to term pregnancy, the risk of LBW was lower in late-term pregnancy (aOR: 0.69 (95% CI: 0.36-0.81)).

**Table 4:** Adverse prenatal outcomes associated with late-term pregnancy OR: odds ratio, CI: confidence interval, aOR: adjusted odds ratio, LBW: low birth weight

Variables	OR (95% CI)	P value	aOR (95% CI)	P value
LBW	0.48 (0.16-0.71)	<0.001	0.69 (0.36-0.81)	<0.001
Macrosomia	3.56 (1.98-5.67)	<0.001	2.24 (1.34-3.01)	<0.001
Meconium amniotic fluid	3.12 (1.78-4.12)	<0.001	2.32 (1.59-3.14)	<0.001
Fetal distress	2.97 (1.12-5.13)	<0.001	2.38 (1.54-2.79)	<0.001

## Discussion

Important pregnancy outcomes, such as neonatal mortality, stillbirth, long-term neurological problems, and maternal mortality, are linked to the length of gestation or the infant’s gestational age at birth [6]. This study confirmed that late-term pregnancy was more likely to be associated with macrosomia, meconium amniotic fluid, and fetal distress; however, serious maternal and neonatal adverse events were similar to term pregnancy. In terms of demographic data, no differences were observed between late-term and term mothers. Among obstetrical factors, parity was the only factor associated with gestational age. The late-term group had a higher prevalence of primiparous mothers. Primiparity is one of the most common identifiable risk factors for the prolongation of pregnancy [7]. The late-term group had a lower rate of diabetes in terms of maternal comorbidities. This is due to the recommendation of diabetic guidelines to terminate pregnancy at 39 weeks of gestation [8].

In our study, late-term mothers were at double risk of meconium amniotic fluid and fetal distress (abnormal fetal heart tracing) than term mothers. The presence of meconium amniotic fluid may represent the normal maturation of the gastrointestinal tract. It may also be present in cases of fetal distress caused by an acute or chronic hypoxic event, which is hard to distinguish [9]. Intrauterine meconium passage in near-term or term fetuses has been linked to fetomaternal stress factors and/or infection, whereas late-term and post-term meconium passage has been linked to gastrointestinal maturation [10]. Although meconium amniotic fluid is associated with an increased risk of fetal distress (abnormal fetal heart tracing) [11], the relationship between specific fetal tracing abnormalities and neonatal outcomes in this context remains unknown [12]. Prolonged decelerations, severe variable decelerations, bradycardia (baseline fetal heart rate (FHR) of 110 beats/minute), and tachycardia (baseline FHR of 160 beats/minute) were found to be independently associated with perinatal mortality and/or neonatal morbidity [13]. In our study, despite the increased risk of meconium amniotic fluid and fetal distress, adverse neonatal outcomes such as asphyxia, need for resuscitation, NICU admission, or neonatal death were not significantly higher compared to term mothers. We were unable to extract data on the type of fetal heart tracing in our study, so we cannot draw any conclusions about the low rate of adverse neonatal outcomes even in the presence of fetal distress. Early management of fetal distress, including the prompt decision for cesarean section, could be one reason.

Based on our findings, the risk of LBW was lower in late-term mothers compared to term pregnant mothers. LBW refers to infants weighing less than 2,500 grams who are either born too soon, i.e., preterm birth, or too small, i.e., fetal growth restriction. LBW is a well-established risk factor for infant mortality and morbidity, as well as a recognized proxy for maternal health [14]. Garcia et al. discovered a significant difference in infant mean birth weights and gestational age; the greater the gestational age, the greater the infant’s weight [15]. On the other hand, late-term pregnancy was strongly associated with macrosomia. Macrosomia is associated with an increased risk of several maternal and neonatal complications. Maternal complications include an increase in the number of cesarean sections performed, extensive perineal lacerations, and severe hemorrhage. Shoulder dystocia, hypoglycemia, respiratory distress, and death are all examples of neonatal complications [16]. Both diabetic and nondiabetic pregnancies had these negative outcomes [17]. However, in our study, despite the higher rate of macrosomia in late-term mothers, the maternal and neonatal adverse events did not increase compared to term mothers. More research is needed to investigate the relationship between newborn weight as an independent factor and adverse prenatal outcomes in late-term pregnancy.

The strength of our study is that our study registers are of high quality and in accordance with childbirth records. We investigated various maternal and neonatal outcomes. Our study was conducted retrospectively, which is still a limitation. The database did not allow for the precise timing of the various events during pregnancy. More data was missing for variables, such as body mass index. However, the fact that the obstetric team entered clinical findings prospectively into the database and there was a large body of evidence available for analysis both increase the reliability of the findings.

## Conclusions

A higher percentage of late-term mothers were primiparous. Diabetes was less common in late-term pregnant mothers due to early termination of pregnancy at 38-39 weeks of gestation. Late-term pregnancy was found to be more likely to be associated with macrosomia, meconium amniotic fluid, and fetal distress, but serious maternal and neonatal adverse events were comparable to term pregnancy. Further research is needed to make better conclusions.
